# Global research landscape and trends of papillary thyroid cancer therapy: a bibliometric analysis

**DOI:** 10.3389/fendo.2023.1252389

**Published:** 2023-09-19

**Authors:** Bo Song, Zheyu Lin, Chuyao Feng, Xu Zhao, Weiping Teng

**Affiliations:** Department of Endocrinology and Metabolism, Institute of Endocrinology, National Health Commission Key Laboratory of Diagnosis and Treatment of Thyroid Diseases, The First Hospital of China Medical University, Shenyang, Liaoning, China

**Keywords:** papillary thyroid carcinoma, therapy, bibliometric analysis, research frontiers, hotspots

## Abstract

**Background:**

Papillary thyroid cancer (PTC) is the most common endocrine malignancy worldwide. The treatment of PTC has attracted extensive attention and discussion from the public and scholars. However, no article has systematically assessed the related literature. Therefore, we conducted a bibliometric and knowledge map analysis to reveal the dynamic scientific developments in the PTC therapy field.

**Methods:**

We retrieved publications related to PTC therapy from the Web of Scientific Core Collection (WoSCC) on May 1, 2023. The bibliometric package in R software, VOSviewer and CiteSpace software were used to analyze countries/regions, institutions, journals, authors, references, and keywords. Then, we systematized and summarized the research landscape, global trends and hot topics of research.

**Results:**

This bibliometric analysis spanned from 2012 to 2022 and involved 18,501 authors affiliated with 3,426 institutions across 87 countries/regions, resulting in the publication of 3,954 papers in 860 academic journals. Notably, the number of publications and citations related to PTC therapy research has exhibited a steady increase over the past decade. China and the United States have emerged as leading contributors in terms of publication count, with the United States also being the most cited country. Furthermore, among the top 10 institutions with the highest number of published papers, half were located in China. Among the journals, Thyroid is ranked first in terms of total publications and citations. The most productive individual author was Miyauchi Akira. While previous research primarily focused on surgery and radioactive iodine therapy, the increasing emphasis on health awareness and advancements in medical technology have led to the emergence of active surveillance, thermal ablation, and genomic analysis as prominent areas of research.

**Conclusion:**

In conclusion, this comprehensive and quantitative bibliometric analysis elucidates the research trends and hotspots within PTC therapy, drawing from a substantial body of publications. This study provides valuable insights into the historical and current landscape of PTC therapy research while also offering guidance for future research directions. This study serves as a valuable resource for researchers and practitioners seeking new avenues of exploration in the field.

## 1 Introduction

Thyroid cancer (TC) has become the most common endocrine neoplasia, as its global incidence has steadily increased in the past three decades (1). Advancements in diagnostic technology have led to a year-on-year increase in the detection rate of TC, which now has the fifth highest incidence among malignant tumors (2). The increasing incidence of TC poses significant global public health concerns. Papillary thyroid carcinoma (PTC) is the most common subtype of thyroid cancer and accounts for approximately 80% of all thyroid cancer cases worldwide (3). Although most PTCs have a relatively good prognosis, approximately 10% of patients may progress to invasive disease, 5% progress to distant metastasis, and approximately 20–30% may relapse (4). The treatment of PTC has evolved over the years, and many new therapeutic options have emerged. Conventional treatments for PTC differ based on the stage of the initial diagnosis, age, and overall health of the patient. Surgery is the primary treatment for PTC, and it can be curative in most cases. The extent of surgery depends on the size of the tumor, lymph node involvement, and risk factors for recurrence. Total thyroidectomy with or without cervical lymph node dissection is recommended for patients with intermediate- and high-risk tumors (5). Radioactive iodine therapy is also recommended in some cases to ablate any remaining thyroid tissue or to treat metastatic disease. In recent years, there have been advancements in targeted therapies for thyroid papillary carcinoma. These therapies target molecular pathways that are deregulated in PTC and are associated with better outcomes in patients with refractory disease. Targeted therapies are typically used in cases where the cancer has spread or has become resistant to other types of treatments (6). Early detection and prompt treatment play a crucial role in improving the prognosis of patients with thyroid papillary carcinoma. Regular check-ups and follow-ups with healthcare providers are essential for long-term disease management. Longitudinal studies have found that active surveillance, which is a monitoring approach investigated for PTC, is a safe alternative to immediate surgery for patients confirmed to have small (<1-1.5 cm) PTCs (7, 8).

In scientific research, publication is a vital index to measure the contribution of scientific research. Bibliometric analysis is a quantitative method used to depict the knowledge structures and developmental trends of a specific field and evaluate research output, productivity, and impact (9). Unlike other major review methods, bibliometric analysis is well suited for the comprehensive evaluation of an entire academic discipline, encompassing thousands of publications. Bibliometric analysis could provide an impartial approach to evaluating the influence of scientific publications through mathematical and statistical methods, thereby aiding in the identification of research gaps and areas that require further study. In recent years, several bibliometric analyses related to thyroid cancer have been published (10–12). However, there is currently no reported information regarding the quantity and quality of research on PTC treatment. Therefore, we aimed to perform bibliometric network analysis to assess the developmental framework, current landscape, and future trends in the field of PTC treatment.

The present bibliometric analysis analyzed original articles directly related to clinical treatment for PTC published from January 1, 2012, to December 31, 2022. A total of 3954 articles were identified. Furthermore, an additional bibliometric analysis was conducted based on these publications to indicate the latest research hotspots. This study aimed to introduce a novel method for analyzing PTC research, offering an unbiased perspective on research trends, identifying influential authors and institutions, and mapping the intellectual structure of the field.

## 2 Materials and methods

### 2.1 Data collection

A database was built to retrieve related literature for bibliometric analysis via the Web of Science (WoS) Core Collection database. To prevent errors resulting from database upgrades, data searches and exports were conducted on May 1, 2023, encompassing articles related to PTC therapy published between January 1, 2012, and December 31, 2022. The search strategy was designed as follows: TS= (“papillary thyroid carcinoma” or “thyroid papillary carcinoma” or “thyroid carcinoma, papillary” or “papillary thyroid cancer” or “thyroid papillary cancer” or “thyroid cancer, papillary”) AND TS= (therapy OR therapies OR treatment). We initially retrieved 5503 records using this search strategy. To minimize potential bias in our analysis, the following refining criteria were applied: (1) timespan: January 1, 2012, to December 31, 2022; (2) document types included article and review; and (3) written in English. Consequently, a total of 3954 articles were obtained for in-depth analysis. Two authors independently extracted the data from the included studies. The flowchart of article inclusion is shown in **Supplementary Figure S1**.

### 2.2 Data analysis

Microsoft Office Excel 2019 software (Microsoft, Redmond, WA, USA) was used to manage data and analyze annual publications. GraphPad Prism 9 software (Dotmatics, San Diego, CA, USA) was used to generate histograms and bubble diagrams. The “bibliometrix” package of R (v4.1.1) is an open-source tool for performing comprehensive science mapping analysis (13). Additionally, the bibliometric analysis utilized two different software tools: CiteSpace [version 6.1. R2 (64-bit)] and VOSviewer (version 1.6.17). CiteSpace is a JAVA-based citation visualization software developed by Chaomei Chen that performs statistical analysis and converts the raw data into a visualization and analysis of literature networks (14). Furthermore, CiteSpace was employed to analyze keywords and references that exhibited significant citation bursts, thus enabling the creation of visualization maps depicting cocited references and keywords. VOSviewer is a powerful software tool for bibliometric mapping developed by Nees Jan van Eck and Ludo Waltman in 2009 (15). It was used to visualize the citation, collaboration, and co-occurrence relationships among journals, references, countries, and institutions. To achieve a comprehensive overview of the research field and ensure result validation, we employed a combination of both software tools. The bibliometric analysis results were subsequently analyzed with descriptive statistics, presented using tables and graphs. To visualize the number of publications in different countries and regions, we utilized Tableau v10.5.0 to create a world map.

## 3 Results

### 3.1 Annual publications

**Figure 1:**
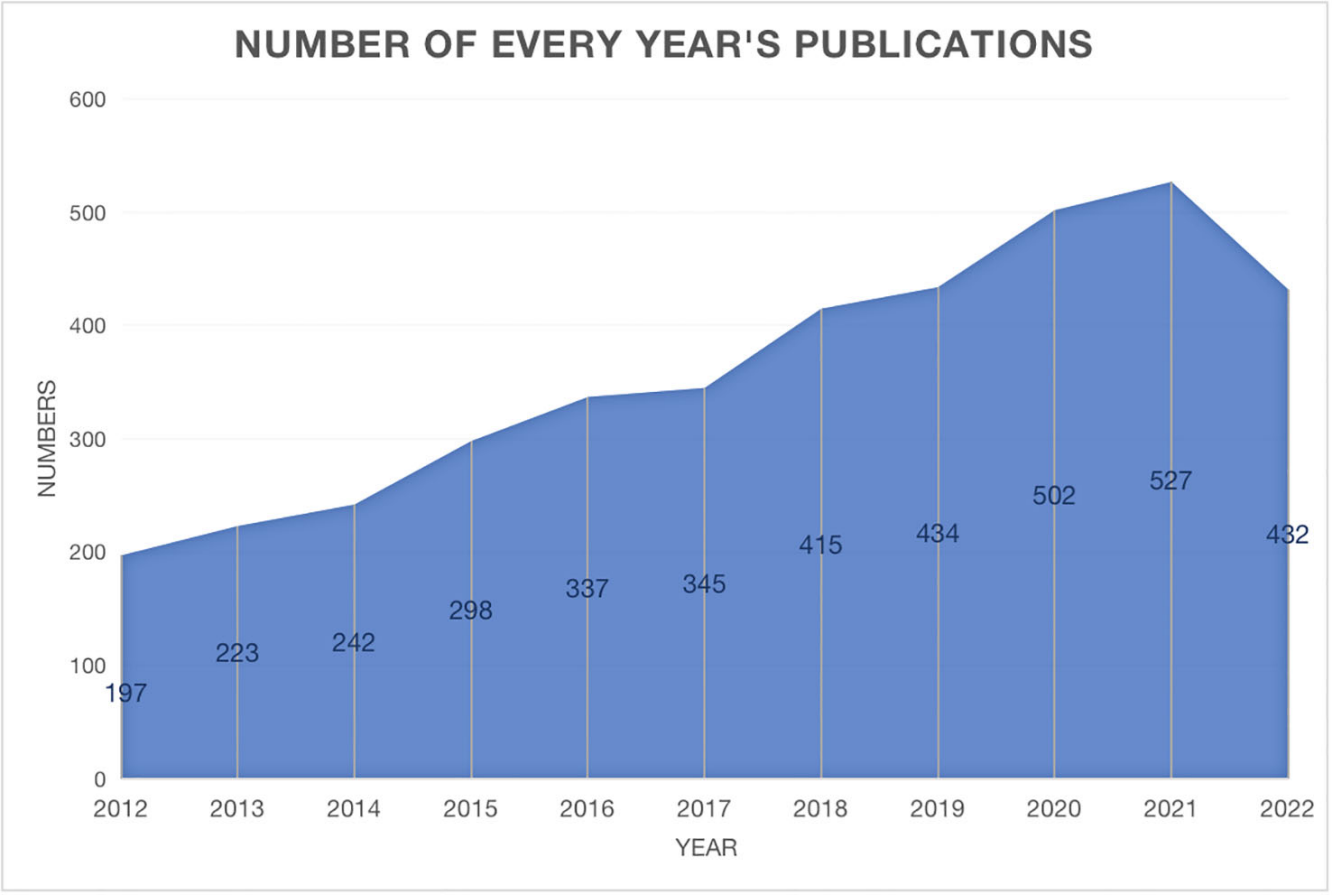
Annual trends of global publication outputs in PTC therapy research from 2012 to 2022.

The annual trend publications associated with therapy for PTC from 2012 to 2022 are presented in **Figure 1**. This study identified a total of 3954 publications meeting the inclusion criteria, including 3468 articles and 486 reviews. The continuous increase in the number of publications over the past decade reflects the increase in attention toward PTC therapy research. The quantity of published documents serves as a crucial indicator of the pace of knowledge updates in this subject and helps to elucidate the trends in the field. When evaluating the number of publications per year, the year with the highest number of publications was 2021 (527, 13.3%). The bibliometric analysis revealed a consistent linear growth trend (R^2 = ^0.8974) in research related to PTC therapy, indicating a growing interest in this field of study (**Supplementary Figure S2**).

### 3.2 Countries

**Figure 2:**
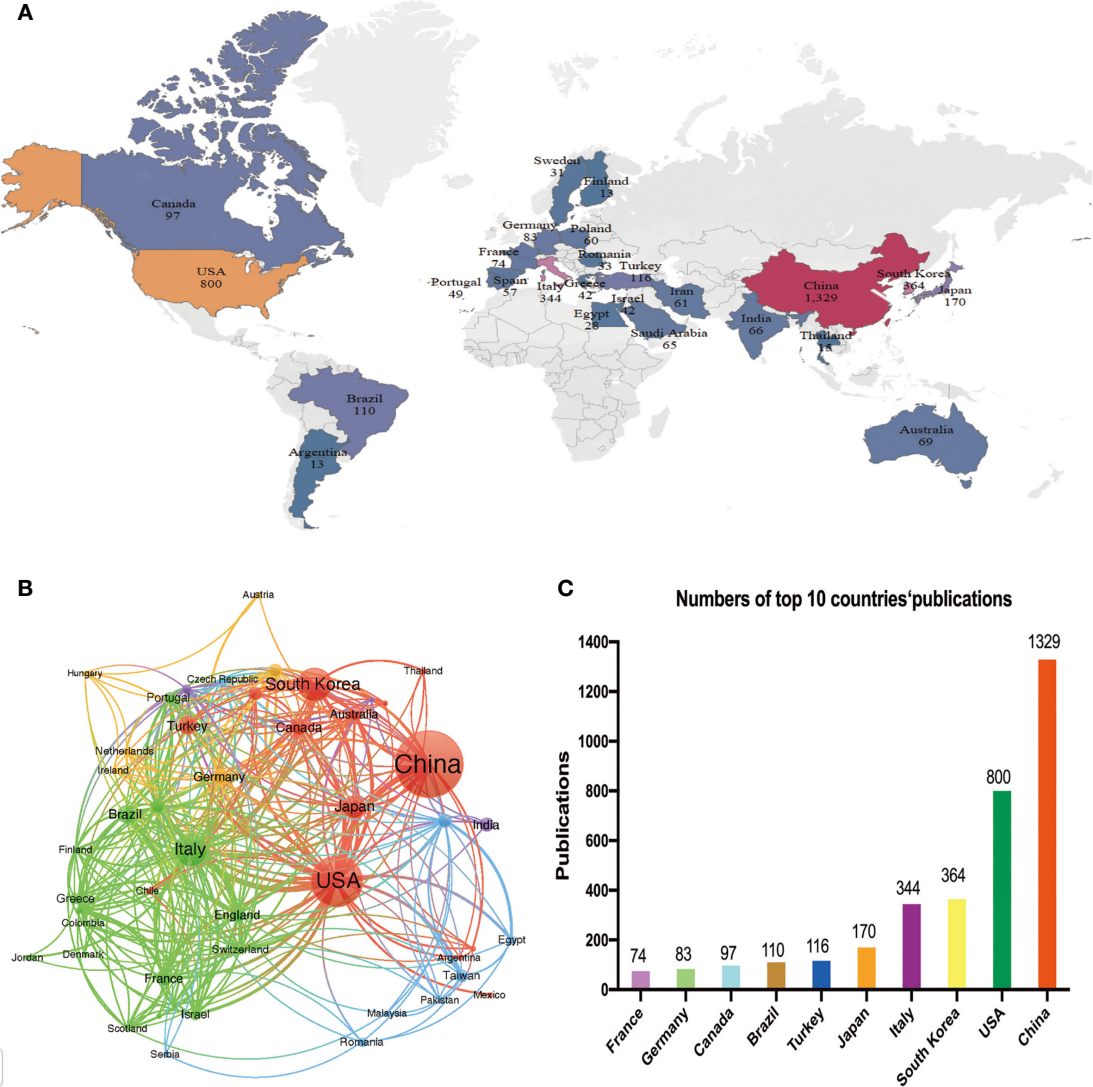
Analysis of the distribution of countries or regions. **(A)** Geographical distribution of the countries/regions in terms of publications. Country name and publication number are shown on the map. **(B)** Network mapping of international collaboration. **(C)** Histogram of the top ten countries with the number of publications.

A total of 87 countries or regions contributed to publications in the field of PTC treatment. **Figure 2A** visualizes the world map of publications in this field. The figure displays the top 25 countries geographically, and the minimum number of documents from a country was 13. Significant achievements were observed in North America, East Asia, and Western Europe, with **Figure 2B** demonstrating strong cooperation among these countries. A coauthorship network was constructed by including 43 countries/regions with a threshold of ten documents, resulting in six clustered groups. Each node in the network represented a country or region, with node size corresponding to the number of publications. The lines connecting the nodes represent cooperation between countries. In addition, **Table 1** and **Figure 2C** present the scientific production of countries.

**Table 1:** The top 10 productive countries/regions in the PTC therapy research field.

Rank	Countries/regions	Publications	Proportion of publications	Citations	Citations per publication
1	China	1329	33.59%	15404	11.59
2	USA	800	20.22%	24835	31.04
3	South Korea	364	9.20%	5694	15.64
4	Italy	344	8.70%	10951	31.83
5	Japan	170	4.30%	3311	19.48
6	Turkey	116	2.93%	685	5.91
7	Brazil	110	2.78%	1657	15.06
8	Canada	97	2.45%	4160	42.89
9	Germany	83	2.10%	1737	20.93
10	France	74	1.87%	1719	23.23

Among the top five countries in terms of productivity, China led with the highest number of papers published (1329, 33.59%), followed by the United States (800, 20.23%), South Korea (364, 9.21%), Italy (344, 8.70%), and Japan (170, 4.30%). Although China ranked first globally in terms of publication volume, its citation/publication rate (11.59) was comparatively low compared to other countries. This suggests that the quality of China’s papers needs further improvement.

### 3.3 Institution analysis

**Figure 3:**
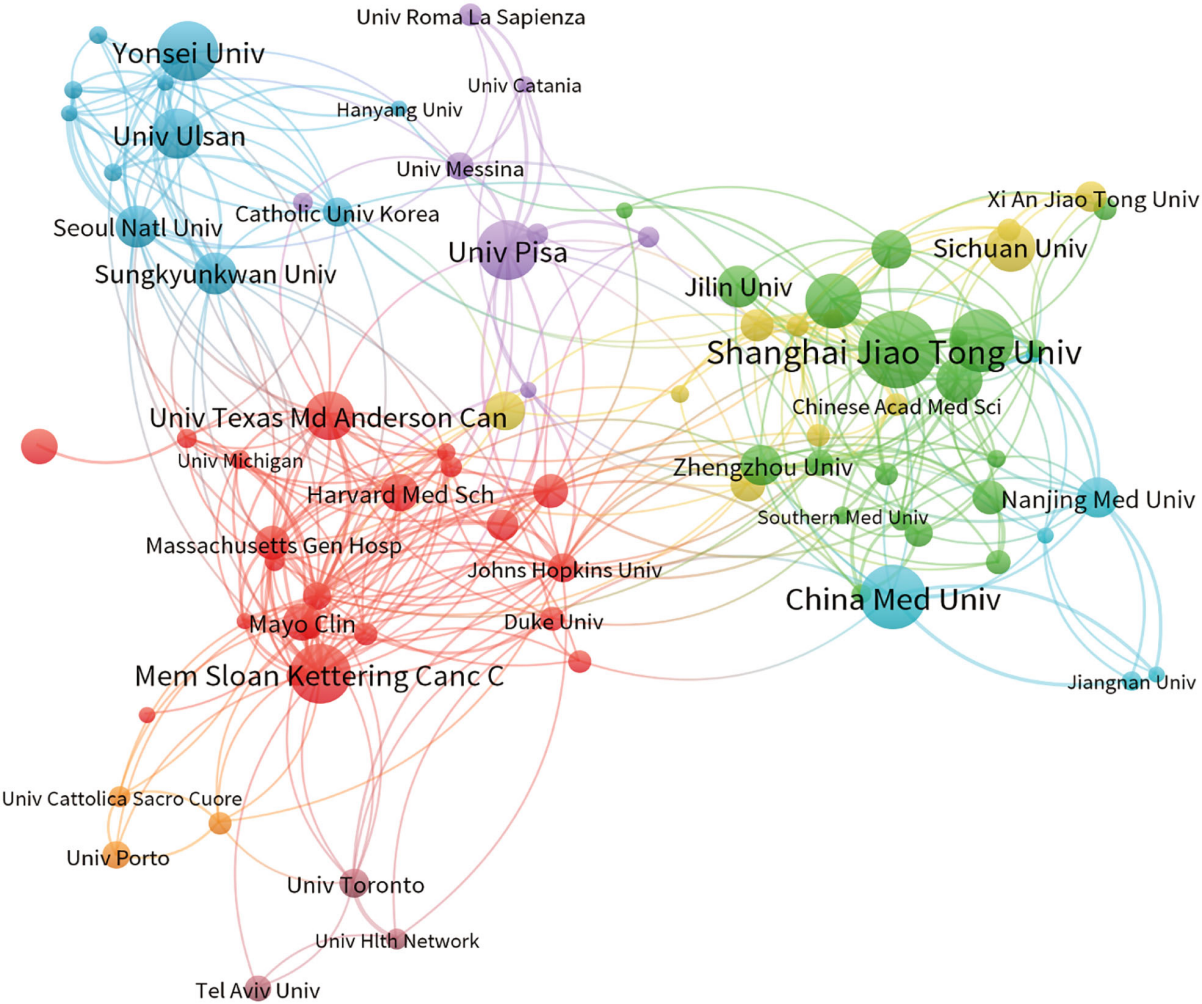
The co-occurrence map of institutions depicts the relationships between them. The size of the nodes corresponds to the number of articles associated with each institution; the thickness of the curves indicates the strength of collaboration. The colors used in the map distinguish various collaboration groups.

The present study involved a total of 3426 institutions, with the top 10 institutions contributing 17.15% of the total papers. **Table 2** lists these top 10 prolific institutions, with half of them located in China and the remaining institutions located in the United States, South Korea, and Italy. Shanghai Jiao Tong University made the highest contribution, with 93 papers and 1396 citations, followed by China Medical University (75 papers, 1161 citations) and Fudan University (74 papers, 947 citations). Among these top ten institutions, the University of Pisa exhibited the highest citation/publication rate (56.63). An analysis of institutional cooperation was conducted to reveal the collaborations between institutions (**Figure 3**). Institutions depicted in the same color demonstrated more active cooperation during the survey. Shanghai Jiao Tong University, University of Pisa, and Memorial Sloan Kettering Cancer Center emerged as central partners. However, there was a notable lack of collaboration among these top research institutions within each region.

**Table 2:** The top 10 productive institutions within PTC therapy research from 2012 to 2022.

Rank	Institutions	Publications	Citations	Country	Citations per publication
1	Shanghai Jiao Tong University	93	1396	China	15.01
2	China Medical University	75	1161	China	15.48
3	Fudan University	74	947	China	12.80
4	Memorial Sloan Kettering Cancer Center	70	3848	The United States	54.97
5	Yonsei University	70	890	Korea	12.71
6	University of Pisa	68	3851	Italy	56.63
7	Zhejiang University	63	571	China	9.06
8	University of Ulsan	57	930	Korea	16.32
9	The University of Texas MD Anderson Cancer Center	55	2179	The United States	39.62
10	Sichuan University	53	376	China	7.09

### 3.4 Journals and cocited journals

**Figure 4:**
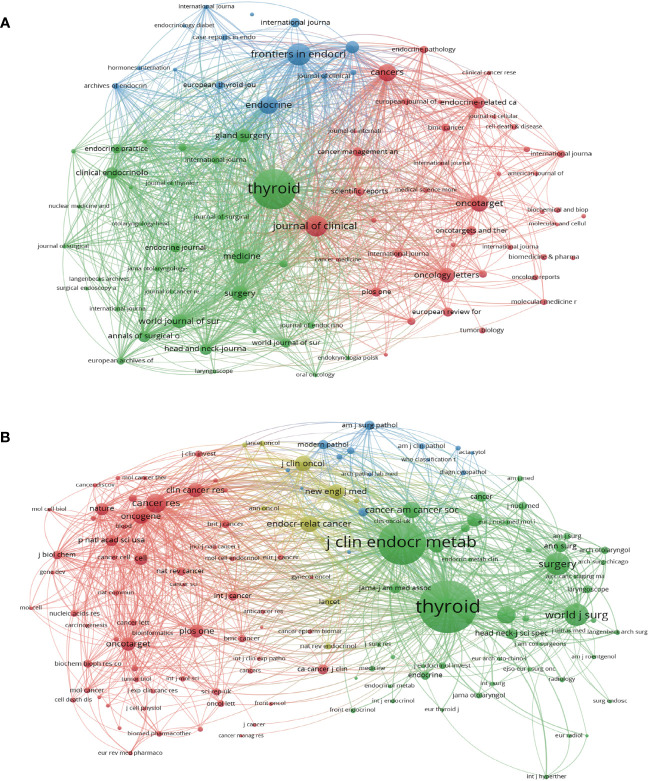
Journals and cocited journals analysis. **(A)** The bibliographic coupling network of journals related to PTC treatment. Each circle in the figure represents a journal, and the size of the circle indicates the number of publications output in that journal. **(B)** The cocitation network visualization of journals with a minimum of 200 citations. The size of the circle indicates the number of citations in that journal.

The 3954 included papers were published in 860 academic journals over the past decade. **Table 3** presents the top 10 productive journals, offering valuable insights into the most prolific and highly cited publications. The bibliographic coupling network of journals related to PTC treatment is shown in **Figure 4A**. Among the 88 journals that published a minimum of 10 papers, Thyroid published the highest number of papers (186), followed by Frontiers in Endocrinology (87 papers) and Journal of Clinical Endocrinology and Metabolism (J CLIN ENDOCR METAB) (81 papers). The impact factor, quartile, and categories were retrieved from the Journal Citation Reports (JCR). Most of the listed journals were categorized under “endocrinology and metabolism” and “surgery.” Except Oncotarget, which was delisted from WoS in 2017, five of the top ten journals were located in JCR quartile one, indicating their high quality. Furthermore, **Figure 4B** shows the results of cocitation analysis of 141 journals with a minimum citation count exceeding 200. Among the top 10 cocited journals, half of them were cited more than 1000 times. Thyroid had the highest total number of citations (6034, IF=6.506), followed by Journal of Clinical Endocrinology and Metabolism (3404, IF=6.134) and Surgery (1307, IF=4.348). The significantly higher citation count of Thyroid compared to other journals indicates its substantial productivity and influence.

**Table 3:** The core journals that published publications in PTC therapy research field.

Rank	Journal	NP	TC	IF2021	SJR 2021	JCR quartile	Categories
1	THYROID	186	6034	6.506	1.673	Q1	ENDOCRINOLOGY & METABOLISM
2	FRONTIERS IN ENDOCRINOLOGY	87	416	6.055	1.375	Q1	ENDOCRINOLOGY & METABOLISM
3	J CLIN ENDOCR METAB	81	3404	6.134	1.746	Q1	ENDOCRINOLOGY & METABOLISM
4	ENDOCRINE	68	802	3.925	0.873	Q3	ENDOCRINOLOGY & METABOLISM
5	CANCERS	64	587	6.575	1.349	Q1	ONCOLOGY
6	ONCOTARGET	60	1272	NA	NA	NA	ONCOLOGY; CELL BIOLOGY
7	MEDICINE	56	619	1.817	0.470	Q3	MEDICINE, GENERAL & INTERNAL
8	SURGERY	55	1307	4.348	1.236	Q1	SURGERY
9	ONCOLOGY LETTERS	53	507	3.111	0.639	Q3	ONCOLOGY
10	WORLD JOURNAL OF SURGERY	50	1255	3.282	0.874	Q2	SURGERY

NP, Number of published; TC, Total citations.

### 3.5 Publication distribution among author analysis

**Figure 5:**
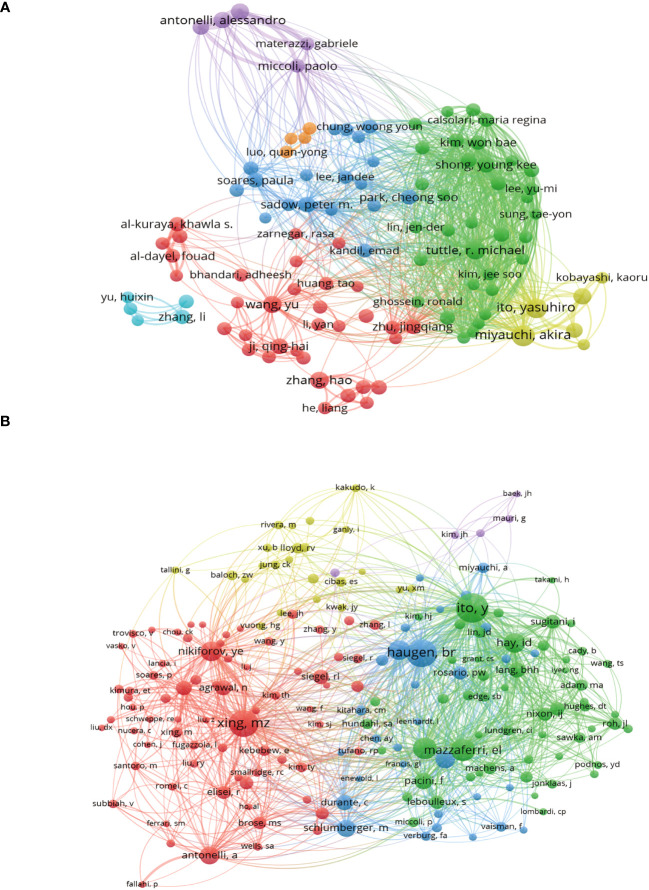
Authors and cocited authors analysis. **(A)** Co-occurrence map of authors. The size of the nodes represents the number of articles. **(B)** Cocited authors analysis map. The size of the nodes represents the number of cocitations.

A total of 18,501 researchers and 48,186 cocited authors made contributions to the 3,954 publications in the field of PTC therapy between 2012 and 2022. **Figure 5A** illustrates coauthor collaboration and the publication output of each author. Collaboration among authors signifies teamwork, although there is less collaboration among teams, resulting in relatively fragmented research. Cocited authors refer to two or more authors who are simultaneously cited through another paper. As depicted in **Figure 5B**, the cocitation analysis revealed that Ito Yasuhiro (1,286), Xing MZ (1,093), and Haugen BR (1,145) had the highest number of cocitations. These findings indicate that the aforementioned authors displayed greater interest in PTC-related research.

**Figure 6:**
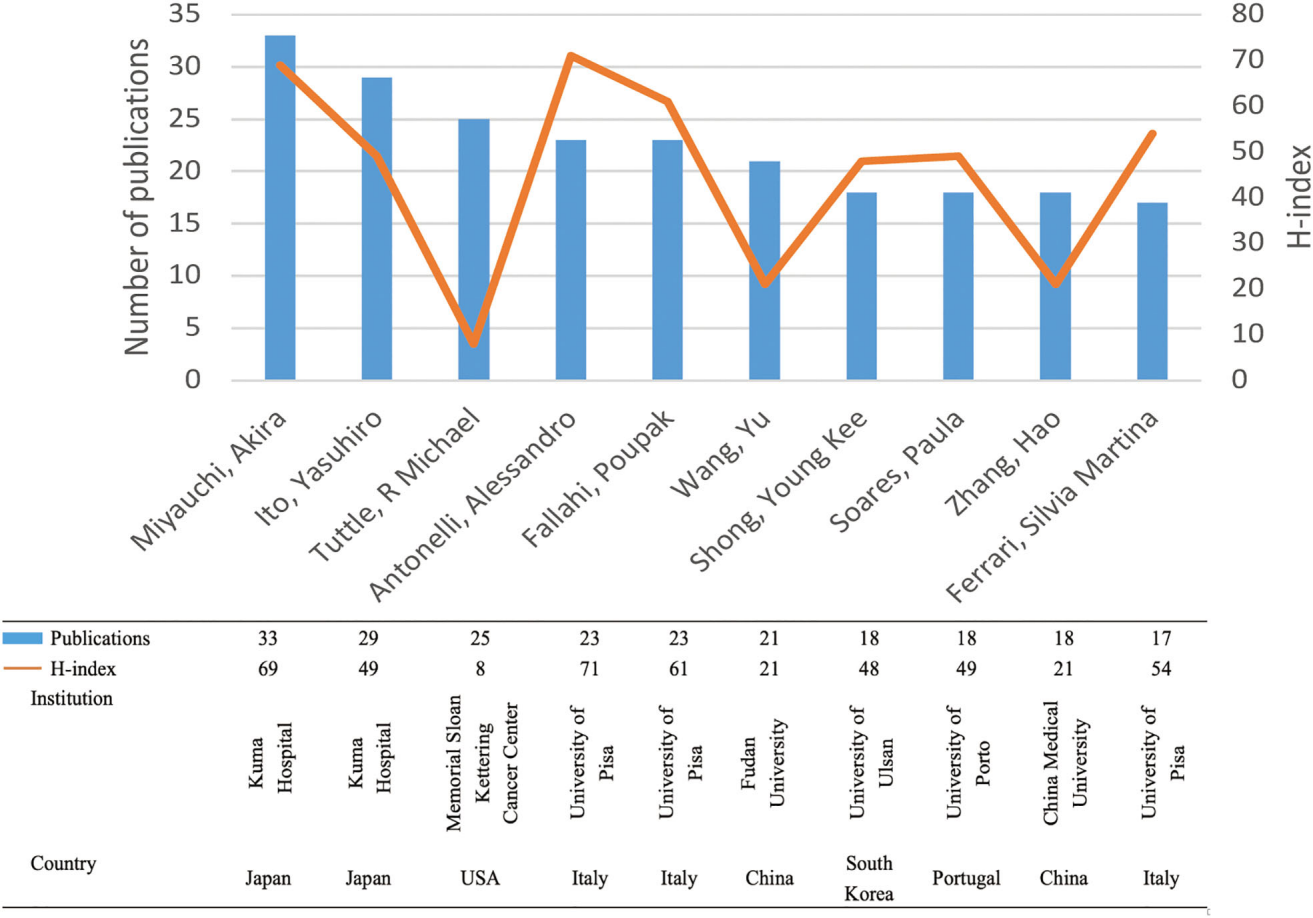
The top 10 productive authors and their H-indexes. Each bar in blue represents the number of publications of each author. Each node in yellow indicates the H-index of each author.

**Figure 6** displays the publication count and H-index of the top 10 productive authors. These ten authors collectively contributed 225 papers (5.69%). Notably, Miyauchi Akira (33), Ito Yasuhiro (29), and Tuttle R Michael (25) emerged as the top three authors in terms of publication volume. The first two authors are affiliated with Kuma Hospital in Japan, an institution specializing in thyroid disorders. Among all authors, Antonelli Alessandro from the University of Pisa had the highest H-index (71), followed by Miyauchi Akira from Kuma Hospital (69) and Fallahi Poupak from the University of Pisa (61).

### References

3.6

Among the 1,690 documents retrieved for this study, the top 10 most cited research articles accounted for a total of 2,012 citations, representing 19.75% of the total citations. **Table 4** presents the top 10 references with the highest cocitations in descending order. Each of these references received more than 90 cocitations. Eight of the publications were from the United States, while the remaining two were from England. The most cited article, titled “2015 American Thyroid Association Management Guidelines for Adult Patients with Thyroid Nodules and Differentiated Thyroid Cancer: The American Thyroid Association Guidelines Task Force on Thyroid Nodules and Differentiated Thyroid Cancer,” was published in 2016 in the journal Thyroid, which is the most cited journal in this field.

**Table 4:** The characteristics of highly cited and the most impact classic articles in PTC therapy field.

Rank	Total Citations	Article title	Journal	Published Year	Country	IF2021
1	842	2015 American Thyroid Association Management Guidelines for Adult Patients with Thyroid Nodules and Differentiated Thyroid Cancer: The American Thyroid Association Guidelines Task Force on Thyroid Nodules and Differentiated Thyroid Cancer	Thyroid	2016	USA	6.506
2	225	Revised American Thyroid Association management guidelines for patients with thyroid nodules and differentiated thyroid cancer	Thyroid	2009	USA	6.506
3	191	Trends in Thyroid Cancer Incidence and Mortality in the United States, 1974-2013	JAMA	2017	USA	157.335
4	174	Integrated genomic characterization of papillary thyroid carcinoma	Cell	2014	USA	66.850
5	106	Global cancer statistics 2018: GLOBOCAN estimates of incidence and mortality worldwide for 36 cancers in 185 countries	CA: A Cancer Journal for Clinicians	2018	USA	286.130
6	98	Thyroid cancer	Lancet	2016	ENGLAND	202.731
7	98	Cancer statistics, 2022	CA: A Cancer Journal for Clinicians	2022	USA	286.130
8	95	Nomenclature Revision for Encapsulated Follicular Variant of Papillary Thyroid Carcinoma: A Paradigm Shift to Reduce Overtreatment of Indolent Tumors	JAMA Oncology	2016	USA	33.006
9	92	The changing incidence of thyroid cancer	Nature Reviews Endocrinology	2016	ENGLAND	47.564
10	91	Current thyroid cancer trends in the United States	JAMA Otolaryngology-Head & Neck Surgery	2014	USA	8.961

**Figure 7:**
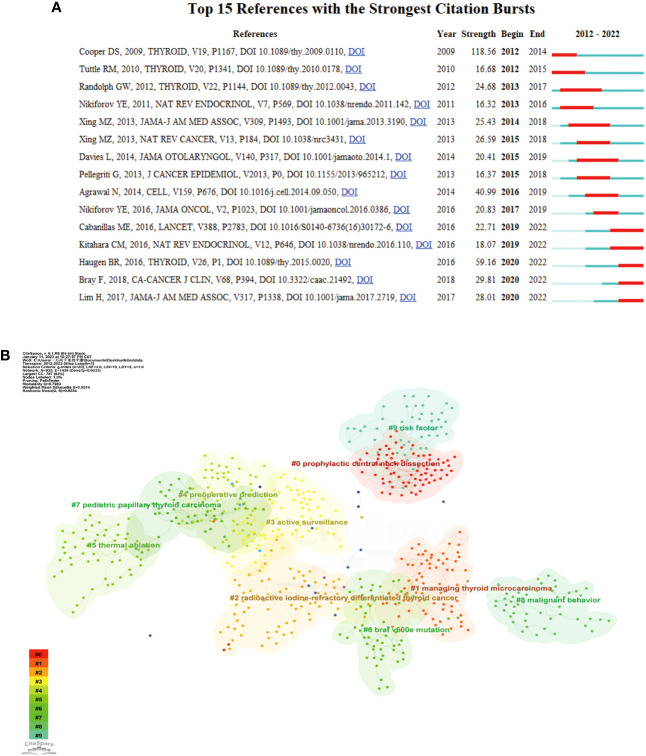
**(A)** Burst analysis of the top 15 references. The blue line represents the period from 2012 to 2022, while the red line plots the periods of each burst keyword. **(B)** Reference clustering map analysis through CiteSpace. A total of 10 categories of references were obtained. The different color blocks represent different reference clusters.

CiteSpace software was employed to conduct burst analysis and cluster analysis of cocited references. In bibliometric analysis, reference bursts can indicate research hotspots in academic fields. **Figure 7A** displays the top 15 cocited references with the strongest citation bursts from 2012 to 2022. The strength of these bursts ranged from 16.32 to 118.56. The blue line segment represents the time interval, while the red line segment indicates the time of frequent citations. The top three references with the most pronounced citation bursts were “Revised American Thyroid Association management guidelines for patients with thyroid nodules and differentiated thyroid cancer” (Strength: 118.56; Publication Year: 2009), “2015 American Thyroid Association Management Guidelines for Adult Patients with Thyroid Nodules and Differentiated Thyroid Cancer” (Strength: 59.16; Publication Year: 2015) and “Integrated genomic characterization of papillary thyroid carcinoma” (Strength: 40.99; Publication Year: 2016).

As depicted in **Figure 7B**, the research field’s topic clustering map identified 10 clusters with significant modularity and silhouette scores (Q = 0.7983; S = 0.9214). These two indicators are crucial for evaluating the effectiveness of graph visualization. Q-values greater than 0.3 and S-values greater than 0.5 indicate a stable and highly convincing clustering structure. The largest cluster, labeled “prophylactic central neck dissection,” was followed by clusters focused on “managing papillary thyroid carcinoma” (cluster #1), “radioactive iodine-refractory differentiated thyroid cancer” (cluster #2), and “active surveillance” (cluster #3). Other important clusters included papillary thyroid carcinoma, radioiodine therapy, Hashimoto’s thyroiditis, dynamic risk stratification, and association management guidelines.

### 3.7 Analysis of keywords and research trends

**Figure 8:**
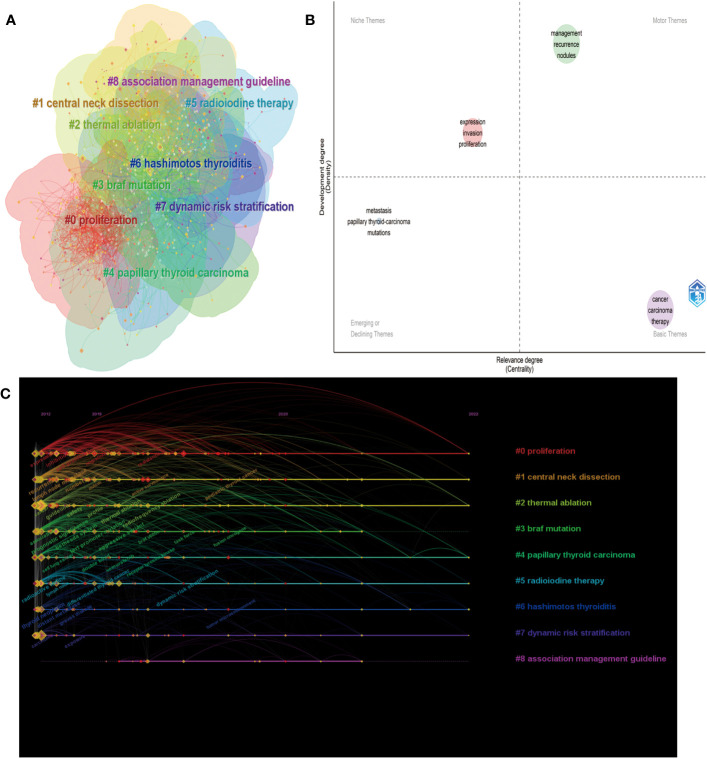
Analysis of keywords. **(A)** Keyword clustering map analysis through CiteSpace. **(B)** The timeline view for keywords related to PTC therapy. **(C)** Thematic map in the PTC therapy field.

Keywords play a crucial role in accurately depicting an article’s topic and reflecting the research frontiers within a specific field of study. In this study, we collected 8,013 keywords from various authors and utilized the CiteSpace cluster function to construct a visual map clustering commonly cited keyword. Through our analysis, we categorized the research documents into nine clusters, as depicted in **Figure 8A**. The module’s Q value was determined to be 0.9122, and the average contour S value was 0.5. The cluster nomenclature effectively represents the study frontiers in the field. In this study, the clusters were autogenerated and labeled using the log-likelihood ratio (LLR) algorithm. The largest cluster, labeled #0, was “proliferation,” followed by “central neck dissection” (cluster #1), “thermal ablation” (cluster #2), and “BRAF mutation” (cluster #3). Other significant clusters included papillary thyroid carcinoma, radioiodine therapy, Hashimoto’s thyroiditis, dynamic risk stratification, and association management guidelines.

To further visualize the thematic map of PTC therapy, we examined the locations of keywords, which were represented by their density and centrality. This representation indicates the evolution of themes (16). The upper right quadrant (Q1) represents crucial motor themes, while the upper left quadrant (Q2) portrays well-established, albeit somewhat isolated, themes. The lower left quadrant (Q3) indicates emerging or declining themes, and the lower right quadrant (Q4) represents basic or transversal themes. As shown in **Figure 8B**, the terms “management” and “recurrence” are located in Q1, demonstrating that these topics are well developed and can structure the research field. The presence of “metastasis” and “mutations” in Q3 is expected, as they represent basic topics in this field. Additionally, the presence of “expression,” “invasion,” and “proliferation” in Q2 implies highly developed internal connections but marginal contributions to advancements in PTC therapy. The timeline viewer (**Figure 8C**) of keywords enables us to observe the evolution of new hotspots and explore the evolutionary trajectory of this field. The keywords were classified into nine clusters, and their evolution can be roughly divided into three periods: the early stage (2012-2015), middle stage (2016-2020), and current stage (2021-2022). Based on the cluster labels and keywords within the clusters, recent research hotspots include “tumor microenvironment,” “fusion oncogene,” “dynamic risk stratification,” and “pediatric thyroid cancer”. **Supplementary Figure S3A** presents the most frequently occurring keywords in the field of PTC therapy from 2012 to 2022. The size of each keyword indicates its importance and frequency of use within the field. To obtain clustering results, we utilized the FoamTrees function of Carrot2 with keywords as the data source, resulting in 34 clusters (**Supplementary Figure S3B**). The larger the area of the foam is, the higher the intensity of research activity. Notable keywords in the clusters include “cells in patients,” “Braf in cancer,” and “Expression in papillary thyroid carcinoma cells.”

## 4 Discussion

### 4.1 Basic information

This study analyzed 3954 publications from 87 countries on PTC therapy over the past decade. The studies were retrieved from the WoSCC database. Bibliometric and visual analysis methods were employed to examine research trends and hotspots in this field from 2012 to 2022. The number of publications on PTC therapy has been steadily increasing each year, with 2021 having the highest volume of literature, thus indicating the growing significance of this topic. China and the United States have emerged as the leading contributors, surpassing other countries/regions in terms of publication count. The United States is the most cited country, and Canada had the highest average number of citations. However, China exhibited a low citation-to-publication ratio, suggesting a need for higher-quality publications. Furthermore, half of the top 10 institutions with the most published papers were located in China, followed by the United States and South Korea.

Regarding journals and cocitations, the most productive journals were Thyroid (186 papers), Frontiers in Endocrinology (87 papers), and Journal of Clinical Endocrinology and Metabolism (81 papers). Thyroid also ranked first in terms of total citations, indicating the inclusion of a substantial number of high-quality articles. Among authors, the most productive individuals were Miyauchi Akira (33), Ito Yasuhiro (29), and Tuttle R Michael (25). The three authors with the highest H-indexes were Antonelli Alessandro (71), Miyauchi Akira (69), and Fallahi Poupak (61), highlighting the superior quality of their papers and establishing them as leading figures in the field of PTC therapy research. Among the top 10 most cited articles, in addition to the guidelines for diagnosing and managing thyroid nodules and cancers, the integrated genomic characterization (17) and the current trend of thyroid cancer (18) also gained researchers’ attention. These articles are all published in JCR Q1 journals, and the quality of the articles is relatively high.

### 4.2 Research trends and hotspots

The analysis and discussion of the aforementioned general information reveal that influential authors and references mostly consist of review articles and clinical guidelines from internationally renowned institutions and journals. By combining co-occurrence, clustering, and burst analysis of keywords and references, we identified continuous and emerging hotspots and research trends in the field of PTC therapy. In the past, research primarily focused on surgery and radioactive iodine therapy. However, with the increasing awareness of people’s health and advancements in medical technology, active surveillance, thermal ablation, and genomic analysis are becoming prominent areas of research.

#### 4.2.1 Central neck dissection

The pros and cons of prophylactic central neck dissection (pCND) have been extensively debated in recent studies. Papillary thyroid carcinoma is a prevalent endocrine malignancy worldwide. While the prognosis for treated PTC is generally favorable, certain patients have a higher risk of recurrence and lymph node metastasis. Surgery remains the most commonly used curative treatment for PTC. Based on preoperative staging and lymph node metastasis, several organizations have advocated for total thyroidectomy (TT) plus pCND as the standard procedure for PTC (5). This recommendation is supported by three primary arguments. First, lymph node metastases are frequently involved in PTC, particularly in the central compartment of the neck (level VI). Second, detecting recurrence or persistence in the paratracheal area is challenging. Last, reoperations in the central neck present an increased risk of recurrent laryngeal nerve injury and hypoparathyroidism. Over time, these three arguments have been substantiated (19, 20).

However, the value of pCND remains a topic of debate among patients without clinical evidence of nodal metastasis, primarily due to its association with an increased incidence of postoperative hypoparathyroidism and potentially permanent hypoparathyroidism (21). Several meta-analyses have reported that the risk of postoperative hypocalcemia is 2.0 to 2.7 times higher following prophylactic CND than after non-CND procedures (22). Moreover, several studies have indicated that the addition of pCND to TT does not significantly reduce the occurrence of future locoregional recurrence (LRR) compared to TT alone (23, 24). In conclusion, preventative neck dissection may be considered an option for certain high-risk patients with papillary thyroid carcinoma; however, its implementation should be carefully evaluated and discussed with a healthcare professional.

#### 4.2.2 Radioiodine therapy

Radioactive iodine (RAI) therapy is a common treatment approach for patients with metastatic PTC (PTC), as it allows for targeted delivery of radiation to cancerous thyroid tissue. This treatment method has been shown to reduce the risk of recurrence and improve survival rates, particularly in cases of differentiated thyroid cancer with lymph node metastasis (25, 26). However, approximately 30% of PTC patients do not initially show iodine uptake for RAI therapy, and others gradually lose response to RAI during treatment. The prognosis for patients with RAI-resistant differentiated thyroid cancer (RAIR-DTC) depends on tumor burden and growth rate, with an overall 10-year survival rate of 10% and a median survival of only 3-5 years (27). The optimal dosage of RAI therapy varies based on individual patient characteristics, considering factors such as residual thyroid tissue size, thyroid-stimulating hormone (TSH) level, and body weight (28). Several randomized prospective studies support the use of lower doses (e.g., 30 mCi) rather than higher doses (e.g., 100 mCi) for managing low-risk thyroid cancer (29). Complications resulting from RAI therapy, including radiation thyroiditis, salivary gland dysfunction, and hematological changes, can negatively impact patients’ quality of life (30). Therefore, a careful assessment of the risks and benefits of RAI therapy is crucial. Potential side effects and the risk of recurrence after treatment should be considered. Close monitoring and regular follow-up care are recommended for patients undergoing radioactive iodine therapy (31).

However, not all PTC patients respond optimally to RAI therapy, and some develop resistance to its effects. Resistance to RAI therapy in PTC is a complex phenomenon with various proposed mechanisms (32). There are several key factors contributing to PTC RAI resistance. Downregulation or loss of sodium/iodide symporter (NIS) expression can limit iodine uptake, reducing the therapeutic effect (33). BRAF V600E Mutation can affect intracellular signaling pathways, including the MAPK pathway, which plays a role in NIS regulation (34). PTCs that dedifferentiate into poorly differentiated or anaplastic thyroid cancer may lose the ability to take up iodine, rendering them resistant to RAI therapy. Factors within the tumor microenvironment, such as hypoxia and inflammation, can influence RAI resistance by altering NIS expression and function (35). Elevated TSH levels can stimulate thyroid cancer cell growth and reduce the effectiveness of RAI therapy. Understanding the underlying mechanisms and implementing strategies to overcome resistance are crucial for improving treatment outcomes. Approaches aimed at increasing NIS expression, combining RAI therapy with targeted therapies and applying personalized medicine have shown promise in some cases to improve response to RAI.

A thought-provoking paper by WR Luo (36) argue that cancer represents a pathological ecosystem, emphasizing the multidimensional spatiotemporal “unity of ecology and evolution.” Within this framework, cancer therapeutic resistance emerges as a dynamic and adaptive process that mirrors the principles of evolution. In the context of PTC, where therapeutic resistance remains a clinical challenge, viewing resistance as an evolutionary process within the cancer ecosystem provides a more comprehensive explanation. The tumor microenvironment, a crucial component of this ecosystem, plays a pivotal role in shaping treatment responses. Factors such as hypoxia, immune interactions, and genetic heterogeneity influence the adaptive evolution of PTC cells, leading to resistance against therapies, including tyrosine kinase inhibitors (TKIs), RAI treatment and immunotherapy. Furthermore, the concept of ecological and evolutionary dynamics in cancer underscores the importance of monitoring treatment responses over time. The emergence of resistant clones within the tumor population highlights the need for ongoing assessment and personalized treatment adjustments.

#### 4.2.3 Thyroid hormone suppression therapy

Thyroid hormone suppression therapy is based on the understanding that thyroid-stimulating hormone (TSH), secreted by the pituitary gland, influences the growth and proliferation of thyroid cancer cells (37). Several studies have demonstrated a direct association between serum TSH concentration and the risk of differentiated thyroid cancer in patients with thyroid nodules, as well as a more aggressive course in those diagnosed with thyroid cancer (38, 39). Stimulation of thyrotropin leads to overexpression of thyroid differentiation genes, such as the sodium iodide symporter, resulting in increased uptake of RAI and enhancing the tumoricidal effect (40). The underlying hypothesis is that after adequate surgery with or without radioactive iodine ablation, long-term thyrotropin (TSH) suppression therapy should be administered to patients with PTC to decrease TSH levels and prevent the recurrence or spread of cancer cells. Studies on the effectiveness of thyroid hormone suppression therapy have produced conflicting results (41–43). There is significant discrepancy regarding the role of thyrotropin suppression in patients with intermediate- or high-risk DTC, with some studies suggesting no significant difference in recurrence rates between patients receiving the therapy and those who do not. Additionally, TSH suppression therapy can cause adverse effects such as osteoporosis and cardiac arrhythmia in certain patients, leading to controversy surrounding its use (44).

#### 4.2.4 Active surveillance

With the widespread use of diagnostic and imaging technologies, the detection of small low-risk papillary thyroid carcinomas (PTCs) has increased, but the corresponding mortality rate has not shown a similar increase (45, 46). Considering the low risk of disease progression in incidentally discovered PTCs, surgical excision may pose more harm than benefit, leading to active discussions about alternative treatment options. The recent guidelines from the American Thyroid Association introduced active surveillance as a strategy for patients with incidentally detected PTCs, providing an alternative to immediate thyroidectomy (5). Active surveillance involves regular monitoring of the cancer through ultrasound and blood tests to assess its growth and progression. Japan was the first country to evaluate the safety of active surveillance for low-risk PTCs. In the early 1990s, two Japanese centers, the Kuma Hospital and the Cancer Institute Hospital (CIH), conducted prospective clinical studies on active surveillance for low-risk (T1aN0M0) PTCs (47, 48). These studies confirmed the safety of active surveillance for papillary thyroid microcarcinomas (<1.0 cm diameter), with 10% to 15% of patients experiencing tumor growth, usually within 5 years.

Several studies have reported promising results for active surveillance. Ito et al. (49) reported that 15.9% of PTCs <1 cm had a growth of 3 mm or more over a 10-year observation period, and new nodal metastases were detected in 3.4% over the same period. Similarly, Tuttle et al. (50) reported growth of ≥3 mm in 2.5% of PTCs up to 1.5 cm in greatest dimension at 2 years and 12.5% at 5 years with a median follow-up of 25 months. These two authors are also among the top-ranked authors in our author analysis, further highlighting the significant attention and interest in active surveillance in the field of PTC treatment. However, importantly, not all cases of thyroid papillary carcinoma are suitable for active surveillance. Patients with high-risk tumors or aggressive forms of the disease should undergo surgical intervention as soon as possible.

#### 4.2.5 Thermal ablations

Some patients with low-risk papillary thyroid microcarcinoma (PTMC) undergoing active surveillance may experience concerns or anxiety regarding the presence of cancer. While addressing this anxiety and unresolved cases of high-risk patients (i.e., patients younger than 40 years) in active surveillance, ultrasound-guided thermal ablation modalities offer an intermediate approach between active surveillance and surgery for patients with low-risk PTCs (51–53). Thermal ablation (TA) technologies such as radiofrequency ablation (RFA), laser ablation (LA), or microwave ablation (MWA) have recently gained interest as minimally invasive treatment modalities for PTCs, showing promising outcomes (54, 55). Importantly, patients categorized as unsuitable for active surveillance should not consider thermal ablation. However, for patients who prefer to avoid surgery, thermal ablation can be considered. The quality of the operator and treatment center plays a crucial role, as the success of the ablation procedure is operator dependent.

Numerous studies have demonstrated favorable outcomes of thermal ablation in PTC. A meta-analysis of data from 17 studies (combined n = 470) reported no instances of local tumor recurrence or distant metastasis after thermal ablation, and the rate of undergoing delayed surgery was only 1.1% (56). Zhang et al. (55) evaluated the efficacy and safety of ultrasound-guided RFA for treating low-risk PTMC. Among the 92 patients included, 10 (41.7%) achieved resolution within 6 months, and 23 (95.8%) achieved resolution within 12 months. No residual or recurrent tumor tissue was detected within the RFA area or in the residual thyroid tissue during follow-up, and no suspicious metastatic lymph nodes were found.

#### 4.2.6 *BRAF*(V600E) mutation

In contemporary times, various molecular markers for tumors have been proposed as potential tools to identify aggressive cases of papillary thyroid carcinoma. These markers include the B-type RAF kinase (BRAF) V600E mutation, RET/PTC rearrangement, and/or RAS mutation (57). The BRAF V600E mutation serves as a prognostic genetic marker that enhances risk stratification and enables tailored management of thyroid cancer patients, even those considered to have low conventional risks (58). Approximately 40-60% of papillary carcinomas exhibit the BRAF V600E mutation, which leads to constitutive activation of the mitogen-activated protein kinase (MAPK) signaling pathway, thereby promoting cell proliferation and survival (59). Research has demonstrated that the presence of the BRAF V600E mutation is associated with aggressive clinicopathological characteristics and a worse prognosis in patients with papillary carcinoma.

A recent meta-analysis comprising 63 studies involving 20,764 patients revealed that the BRAF (V600E) mutation in PTC is associated with lymph node metastasis, extrathyroidal extension, higher TNM stage, recurrence, and reduced overall survival (60). Xing et al. (61) conducted a retrospective multicenter study involving 1849 individuals with PTC from around the world. Their findings demonstrated a significant association between the presence of the BRAF V600E mutation and increased cancer-related mortality among PTC patients. Moreover, the BRAF V600E mutation has been linked to resistance to radioiodine therapy, which is the standard care for differentiated thyroid cancer patients (62). Therefore, further investigations are necessary to assess the prognostic and therapeutic implications of BRAF V600E status in PTC.

#### 4.2.7 Other gene mutations

Certainly, in addition to the well-documented BRAF(V600E) mutation, PTC exhibits a spectrum of genetic alterations and mutations that contribute to its pathogenesis and clinical heterogeneity. Notable genetic mutations and alterations in PTC include RAS mutations, RET/PTC rearrangements, TP53 mutations, TERT promoter mutations, PIK3CA mutations, EIF1AX mutations, and non-V600E mutations in the BRAF gene (63). Second in prevalence to BRAF mutations in thyroid cancer are RAS mutations. RAS mutations, specifically in NRAS and HRAS, have been identified in a subset of PTC cases, leading to the activation of the mitogen-activated protein kinase (MAPK) signaling pathway and facilitating tumor growth and progression (64).RET/PTC rearrangements, which result from chromosomal rearrangements involving the RET proto-oncogene, lead to constitutive activation of the RET receptor tyrosine kinase and are prevalent in PTC, particularly in radiation-induced cases (65). TP53 mutations are associated with more aggressive forms of PTC and are involved in resistance to radioiodine therapy. They are relatively rare but have been reported in a subset of cases. TERT Promoter Mutations: Mutations in the telomerase reverse transcriptase (TERT) promoter are often seen in aggressive PTC and are associated with increased tumor aggressiveness and poor prognosis (66). These genetic alterations contribute to the heterogeneity of PTC and can have implications for prognosis and treatment strategies. It’s important to note that the prevalence of these mutations can vary among PTC cases and may have different clinical implications.

### 4.3 Limitations

To our knowledge, this study represents the first bibliometric analysis and visualization of research on therapy for PTC. However, our study does possess inherent limitations associated with bibliometric analysis. First, this study only included literature retrieved from the WoS database, and papers not included in this database were omitted. Although the WoS database is widely recognized, extensively used, and comprehensive in bibliometrics (67), the results obtained reflect the overall trends. Second, our study focused on retrieving literature related to PTC therapy published between 2012 and 2022 to capture the latest research trends and landscape in this field, thereby excluding earlier papers. Last, a certain degree of language bias was present, as our analysis only included English-language publications.

## 5 Conclusion

In conclusion, this study provides a comprehensive bibliometric analysis and visualization of the research area pertaining to therapy for PTC. We conducted a bibliometric analysis by retrieving literature relevant to the topic from the Web of Science (WoS) Core Collection database, spanning the years 2012 to 2022. The retrieved data were analyzed using CiteSpace and VOSviewer software, allowing us to examine the field’s development across various aspects, including countries/regions, institutions, journals, authors, references, and keywords. Through this analysis, we identified current hotspots in the field and explored future directions. The most prominent research topics in the field of papillary thyroid carcinoma (PTC) therapy were found to be active surveillance, thermal ablation, and BRAF (V600E) mutation. Overall, our study provides insights into the historical and current trends in PTC therapy research, offering valuable guidance for both researchers and practitioners seeking new avenues of exploration.

## Data availability statement

The original contributions presented in the study are included in the article/**Supplementary Material**. Further inquiries can be directed to the corresponding author.

## Author contributions

BS is the first author of this study. WT is the corresponding author supervising this work. BS managed the case and drafted the manuscript. ZL, CF and XZ provided major technical support. BS, ZL, CF and XZ assisted in literature review and organizing data from the literature. WT and reviewed the manuscript. All authors contributed to the article and approved the submitted version.

## References

[1] Vaccarella S Dal Maso L Laversanne M Bray F Plummer M Franceschi S. The impact of diagnostic changes on the rise in thyroid cancer incidence: A population-based study in selected high-resource countries. Thyroid (2015) 25(10):1127–36. doi: 10.1089/thy.2015.0116 26133012

[2] Rahib L Smith B Aizenberg R Rosenzweig A Fleshman J Matrisian L. Projecting cancer incidence and deaths to 2030: the unexpected burden of thyroid, liver, and pancreas cancers in the United States. Cancer Res (2014) 74(11):2913–21. doi: 10.1158/0008-5472.CAN-14-0155 24840647

[3] Vaccarella S Franceschi S Bray F Wild CP Plummer M Dal Maso L. Worldwide thyroid-cancer epidemic? The increasing impact of overdiagnosis. N Engl J Med (2016) 375(7):614–7. doi: 10.1056/NEJMp1604412 27532827

[4] Chowdhury S Veyhl J Jessa F Polyakova O Alenzi A MacMillan C. Programmed death-ligand 1 overexpression is a prognostic marker for aggressive papillary thyroid cancer and its variants. Oncotarget (2016) 7(22):32318–28. doi: 10.18632/oncotarget.8698 PMC5078015 27086918

[5] Haugen BR Alexander EK Bible KC Doherty GM Mandel SJ Nikiforov YE. 2015 American thyroid association management guidelines for adult patients with thyroid nodules and differentiated thyroid cancer: the American thyroid association guidelines task force on thyroid nodules and differentiated thyroid cancer. Thyroid (2016) 26(1):1–133. doi: 10.1089/thy.2015.0020 26462967 PMC4739132

[6] Carneiro RM Carneiro BA Agulnik M Kopp PA Giles FJ. Targeted therapies in advanced differentiated thyroid cancer. Cancer Treat Rev (2015) 41(8):690–8. doi: 10.1016/j.ctrv.2015.06.002 26105190

[7] Ito Y Miyauchi A Oda H. Low-risk papillary microcarcinoma of the thyroid: A review of active surveillance trials. Eur J Surg Oncol (2018) 44(3):307–15. doi: 10.1016/j.ejso.2017.03.004 28343733

[8] Miyauchi A. Clinical trials of active surveillance of papillary microcarcinoma of the thyroid. World J Surg (2016) 40(3):516–22. doi: 10.1007/s00268-015-3392-y PMC4746213 26744340

[9] Donthu N Kumar S Mukherjee D Pandey N Lim WM. How to conduct a bibliometric analysis: An overview and guidelines. J Business Res (2021) 133:285–96. doi: 10.1016/j.jbusres.2021.04.070

[10] Chen K Wang Z Sun W Zhang D Zhang T He L. Bibliometric insights in advances of papillary thyroid microcarcinoma: Research situation, hot points, and global trends. Front Endocrinol (Lausanne) (2022) 13:949993. doi: 10.3389/fendo.2022.949993 36004350 PMC9393698

[11] Zhang Z Yao L Wang W Jiang B Xia F Li X. A bibliometric analysis of 34,692 publications on thyroid cancer by machine learning: how much has been done in the past three decades? Front Oncol (2021) 11:673733. doi: 10.3389/fonc.2021.673733 34722236 PMC8551832

[12] Wang H Yu Y Wang K Sun H. Bibliometric insights in advances of anaplastic thyroid cancer: research landscapes, turning points, and global trends. Front Oncol (2021) 11:769807. doi: 10.3389/fonc.2021.769807 34900720 PMC8652235

[13] Aria M Cuccurullo C. bibliometrix: An R-tool for comprehensive science mapping analysis. J Informetrics (2017) 11(4):959–75. doi: 10.1016/j.joi.2017.08.007

[14] Chen C. Searching for intellectual turning points: progressive knowledge domain visualization. Proc Natl Acad Sci USA (2004) 101 Suppl 1(Suppl 1):5303–10. doi: 10.1073/pnas.0307513100 PMC387312 14724295

[15] van Eck NJ Waltman L. Software survey: VOSviewer, a computer program for bibliometric mapping. Scientometrics (2010) 84(2):523–38. doi: 10.1007/s11192-009-0146-3 PMC2883932 20585380

[16] Monteagudo-Fernandez J Gomez-Carrasco CJ Chaparro-Sainz A Heritage Education and Research in Museums. Conceptual, intellectual and social structure within a knowledge domain (2000-2019). Sustainability (2021) 13(12). doi: 10.3390/su13126667

[17] Agrawal N Akhbani R Aksoy BA Ally A Arachchi H Asa SL. Integrated genomic characterization of papillary thyroid carcinoma. Cell (2014) 159(3):676–90. doi: 10.1016/j.cell.2014.09.050 PMC4243044 25417114

[18] Kitahara CM Sosa JA. The changing incidence of thyroid cancer. Nat Rev Endocrinol (2016) 12(11):646–53. doi: 10.1038/nrendo.2016.110 PMC10311569 27418023

[19] Onkendi EO McKenzie TJ Richards ML Farley DR Thompson GB Kasperbauer JL. Reoperative experience with papillary thyroid cancer. World J Surg (2014) 38(3):645–52. doi: 10.1007/s00268-013-2379-9 24305931

[20] Urken ML Milas M Randolph GW Tufano R Bergman D Bernet V. Management of recurrent and persistent metastatic lymph nodes in well-differentiated thyroid cancer: a multifactorial decision-making guide for the Thyroid Cancer Care Collaborative. Head Neck (2015) 37(4):605–14. doi: 10.1002/hed.23615 24436291

[21] Cooper DS Doherty GM Haugen BR Kloos RT Lee SL Mandel SJ. Revised American Thyroid Association management guidelines for patients with thyroid nodules and differentiated thyroid cancer. Thyroid (2009) 19(11):1167–214. doi: 10.1089/thy.2009.0110 19860577

[22] Wang TS Cheung K Farrokhyar F Roman SA Sosa JA. A meta-analysis of the effect of prophylactic central compartment neck dissection on locoregional recurrence rates in patients with papillary thyroid cancer. Ann Surg Oncol (2013) 20(11):3477–83. doi: 10.1245/s10434-013-3125-0 23846784

[23] Zetoune T Keutgen X Buitrago D Aldailami H Shao H Mazumdar M. Prophylactic central neck dissection and local recurrence in papillary thyroid cancer: a meta-analysis. Ann Surg Oncol (2010) 17(12):3287–93. doi: 10.1245/s10434-010-1137-6 20596784

[24] Shan CX Zhang W Jiang DZ Zheng XM Liu S Qiu M. Routine central neck dissection in differentiated thyroid carcinoma: a systematic review and meta-analysis. Laryngoscope (2012) 122(4):797–804. doi: 10.1002/lary.22162 22294492

[25] Jacobs D Breen CT Pucar D Holt EH Judson BL Mehra S. Changes in population-level and institutional-level prescribing habits of radioiodine therapy for papillary thyroid cancer. Thyroid (2021) 31(2):272–9. doi: 10.1089/thy.2020.0237 32811347

[26] Schlumberger M Brose M Elisei R Leboulleux S Luster M Pitoia F. Definition and management of radioactive iodine-refractory differentiated thyroid cancer. Lancet Diabetes Endocrinol (2014) 2(5):356–8. doi: 10.1016/S2213-8587(13)70215-8 24795243

[27] Brose MS Smit J Capdevila J Elisei R Nutting C Pitoia F. Regional approaches to the management of patients with advanced, radioactive iodine-refractory differentiated thyroid carcinoma. Expert Rev Anticancer Ther (2012) 12(9):1137–47. doi: 10.1586/era.12.96 23098114

[28] Giovanella L Avram AM Clerc J Hindié E Taïeb D Verburg FA. Postoperative serum thyroglobulin and neck ultrasound to drive decisions about iodine-131 therapy in patients with differentiated thyroid carcinoma: an evidence-based strategy? Eur J Nucl Med Mol Imaging (2018) 45(12):2155–8. doi: 10.1007/s00259-018-4110-4 30062605

[29] Schlumberger M Catargi B Borget I Deandreis D Zerdoud S Bridji B. Strategies of radioiodine ablation in patients with low-risk thyroid cancer. N Engl J Med (2012) 366(18):1663–73. doi: 10.1056/NEJMoa1108586 22551127

[30] Goswami S Peipert BJ Mongelli MN Kurumety SK Helenowski IB Yount SE. Clinical factors associated with worse quality-of-life scores in United States thyroid cancer survivors. Surgery (2019) 166(1):69–74. doi: 10.1016/j.surg.2019.01.034 30898373

[31] Carty SE Cooper DS Doherty GM Duh QY Kloos RT Mandel SJ. Consensus statement on the terminology and classification of central neck dissection for thyroid cancer. Thyroid (2009) 19(11):1153–8. doi: 10.1089/thy.2009.0159 19860578

[32] Liu Y Wang J Hu X Pan Z Xu T Xu J. Radioiodine therapy in advanced differentiated thyroid cancer: Resistance and overcoming strategy. Drug Resist Updat (2023) 68:100939. doi: 10.1016/j.drup.2023.100939 36806005

[33] Spitzweg C Morris JC. The sodium iodide symporter: its pathophysiological and therapeutic implications. Clin Endocrinol (Oxf) (2002) 57(5):559–74. doi: 10.1046/j.1365-2265.2002.01640.x 12390328

[34] Xing M Alzahrani AS Carson KA Shong YK Kim TY Viola D. Association between BRAF V600E mutation and recurrence of papillary thyroid cancer. J Clin Oncol (2015) 33(1):42–50. doi: 10.1200/JCO.2014.56.8253 25332244 PMC4268252

[35] Jiang L Zhan Y Gu Y Ye Y Cheng Y Shi H. Changes of regulatory T and B cells in patients with papillary thyroid carcinoma after 131I radioablation: a preliminary study. BioMed Res Int (2013) 2013:683768. doi: 10.1155/2013/683768 24350284 PMC3856126

[36] Luo W. Nasopharyngeal carcinoma ecology theory: cancer as multidimensional spatiotemporal "unity of ecology and evolution" pathological ecosystem. Theranostics (2023) 13(5):1607–31. doi: 10.7150/thno.82690 PMC10086202 37056571

[37] McLeod DS. Thyrotropin in the development and management of differentiated thyroid cancer. Endocrinol Metab Clin North Am (2014) 43(2):367–83. doi: 10.1016/j.ecl.2014.02.012 24891167

[38] Haymart MR Repplinger DJ Leverson GE Elson DF Sippel RS Jaume JC. Higher serum thyroid stimulating hormone level in thyroid nodule patients is associated with greater risks of differentiated thyroid cancer and advanced tumor stage. J Clin Endocrinol Metab (2008) 93(3):809–14. doi: 10.1210/jc.2007-2215 PMC2266959 18160464

[39] McLeod DS Cooper DS Ladenson PW Ain KB Brierley JD Fein HG. Prognosis of differentiated thyroid cancer in relation to serum thyrotropin and thyroglobulin antibody status at time of diagnosis. Thyroid (2014) 24(1):35–42. doi: 10.1089/thy.2013.0062 23731273 PMC3887423

[40] Derwahl M Broecker M Kraiem Z. Clinical review 101: Thyrotropin may not be the dominant growth factor in benign and Malignant thyroid tumors. J Clin Endocrinol Metab (1999) 84(3):829–34. doi: 10.1210/jcem.84.3.5519 10084556

[41] Ebina A Sugitani I Fujimoto Y Yamada K. Risk-adapted management of papillary thyroid carcinoma according to our own risk group classification system: is thyroid lobectomy the treatment of choice for low-risk patients? Surgery (2014) 156(6):1579–88. doi: 10.1016/j.surg.2014.08.060 25262223

[42] Carhill AA Litofsky DR Ross DS Jonklaas J Cooper DS Brierley JD. Long-term outcomes following therapy in differentiated thyroid carcinoma: NTCTCS registry analysis 1987-2012. J Clin Endocrinol Metab (2015) 100(9):3270–9. doi: 10.1210/JC.2015-1346 PMC5393522 26171797

[43] Park S Kim WG Han M Jeon MJ Kwon H Kim M. Thyrotropin suppressive therapy for low-risk small thyroid cancer: A propensity score-matched cohort study. Thyroid (2017) 27(9):1164–70. doi: 10.1089/thy.2017.0177 28699428

[44] Wang LY Smith AW Palmer FL Tuttle RM Mahrous A Nixon IJ. Thyrotropin suppression increases the risk of osteoporosis without decreasing recurrence in ATA low- and intermediate-risk patients with differentiated thyroid carcinoma. Thyroid (2015) 25(3):300–7. doi: 10.1089/thy.2014.0287 PMC6916125 25386760

[45] Schuster-Bruce J Jani C Goodall R Kim D Hughes W Salciccioli JD. A comparison of the burden of thyroid cancer among the european union 15+ Countries, 1990-2019: estimates from the global burden of disease study. JAMA Otolaryngol Head Neck Surg (2022) 148(4):350–9. doi: 10.1001/jamaoto.2021.4549 PMC8914910 35266977

[46] Udelsman R Zhang Y. The epidemic of thyroid cancer in the United States: the role of endocrinologists and ultrasounds. Thyroid (2014) 24(3):472–9. doi: 10.1089/thy.2013.0257 PMC3949447 23937391

[47] Sugitani I Toda K Yamada K Yamamoto N Ikenaga M Fujimoto Y. Three distinctly different kinds of papillary thyroid microcarcinoma should be recognized: our treatment strategies and outcomes. World J Surg (2010) 34(6):1222–31. doi: 10.1007/s00268-009-0359-x 20066418

[48] Ito Y Uruno T Nakano K Takamura Y Miya A Kobayashi K. An observation trial without surgical treatment in patients with papillary microcarcinoma of the thyroid. Thyroid (2003) 13(4):381–7. doi: 10.1089/105072503321669875 12804106

[49] Ito Y Miyauchi A Inoue H Fukushima M Kihara M Higashiyama T. An observational trial for papillary thyroid microcarcinoma in Japanese patients. World J Surg (2010) 34(1):28–35. doi: 10.1007/s00268-009-0303-0 20020290

[50] Tuttle RM Fagin JA Minkowitz G Wong RJ Roman B Patel S. Natural history and tumor volume kinetics of papillary thyroid cancers during active surveillance. JAMA Otolaryngol Head Neck Surg (2017) 143(10):1015–20. doi: 10.1001/jamaoto.2017.1442 PMC5710258 28859191

[51] Zhang L Zhou W Zhan W Peng Y Jiang S Xu S. Percutaneous laser ablation of unifocal papillary thyroid microcarcinoma: utility of conventional ultrasound and contrast-enhanced ultrasound in assessing local therapeutic response. World J Surg (2018) 42(8):2476–84. doi: 10.1007/s00268-018-4500-6 29488064

[52] Teng DK Li HQ Sui GQ Lin YQ Luo Q Fu P. Preliminary report of microwave ablation for the primary papillary thyroid microcarcinoma: a large-cohort of 185 patients feasibility study. Endocrine (2019) 64(1):109–17. doi: 10.1007/s12020-019-01868-2 30771153

[53] Teng D Sui G Liu C Wang Y Xia Y Wang H. Long-term efficacy of ultrasound-guided low power microwave ablation for the treatment of primary papillary thyroid microcarcinoma: a 3-year follow-up study. J Cancer Res Clin Oncol (2018) 144(4):771–9. doi: 10.1007/s00432-018-2607-7 PMC11813516 29427209

[54] Zhou W Jiang S Zhan W Zhou J Xu S Zhang L. Ultrasound-guided percutaneous laser ablation of unifocal T1N0M0 papillary thyroid microcarcinoma: Preliminary results. Eur Radiol (2017) 27(7):2934–40. doi: 10.1007/s00330-016-4610-1 27853812

[55] Zhang M Luo Y Zhang Y Tang J. Efficacy and safety of ultrasound-guided radiofrequency ablation for treating low-risk papillary thyroid microcarcinoma: A prospective study. Thyroid (2016) 26(11):1581–7. doi: 10.1089/thy.2015.0471 27445090

[56] Cho SJ Baek JH Chung SR Choi YJ Lee JH. Thermal ablation for small papillary thyroid cancer: A systematic review. Thyroid (2019) 29(12):1774–83. doi: 10.1089/thy.2019.0377 31739738

[57] Yarchoan M LiVolsi VA Brose MS. BRAF mutation and thyroid cancer recurrence. J Clin Oncol (2015) 33(1):7–8. doi: 10.1200/JCO.2014.59.3657 25422487

[58] Xing M Haugen BR Schlumberger M. Progress in molecular-based management of differentiated thyroid cancer. Lancet (2013) 381(9871):1058–69. doi: 10.1016/S0140-6736(13)60109-9 PMC3931461 23668556

[59] Davies H Bignell GR Cox C Stephens P Edkins S Clegg S. Mutations of the BRAF gene in human cancer. Nature (2002) 417(6892):949–54. doi: 10.1038/nature00766 12068308

[60] Liu C Chen T Liu Z. Associations between BRAF(V600E) and prognostic factors and poor outcomes in papillary thyroid carcinoma: a meta-analysis. World J Surg Oncol (2016) 14(1):241. doi: 10.1186/s12957-016-0979-1 27600854 PMC5012084

[61] Xing M Alzahrani AS Carson KA Viola D Elisei R Bendlova B. Association between BRAF V600E mutation and mortality in patients with papillary thyroid cancer. JAMA (2013) 309(14):1493–501. doi: 10.1001/jama.2013.3190 PMC3791140 23571588

[62] Ge J Wang J Wang H Jiang X Liao Q Gong Q. The BRAF V600E mutation is a predictor of the effect of radioiodine therapy in papillary thyroid cancer. J Cancer (2020) 11(4):932–9. doi: 10.7150/jca.33105 PMC6959026 31949496

[63] Park JY Kim WY Hwang TS Lee SS Kim H Han HS. BRAF and RAS mutations in follicular variants of papillary thyroid carcinoma. Endocr Pathol (2013) 24(2):69–76. doi: 10.1007/s12022-013-9244-0 23625203

[64] Nikiforova MN Kimura ET Gandhi M Biddinger PW Knauf JA Basolo F. BRAF mutations in thyroid tumors are restricted to papillary carcinomas and anaplastic or poorly differentiated carcinomas arising from papillary carcinomas. J Clin Endocrinol Metab (2003) 88(11):5399–404. doi: 10.1210/jc.2003-030838 14602780

[65] Santoro M Carlomagno F Melillo RM Billaud M Vecchio G Fusco A. Molecular mechanisms of RET activation in human neoplasia. J Endocrinol Invest (1999) 22(10):811–9. doi: 10.1007/BF03343650 10614534

[66] Gröbner SN Worst BC Weischenfeldt J Buchhalter I Kleinheinz K Rudneva VA. The landscape of genomic alterations across childhood cancers. Nature (2018) 555(7696):321–7. doi: 10.1038/nature25480 29489754

[67] Zhang Y Wang Y Chen J Cheng Q Zhang B Hao L. The top 100 cited articles in osteonecrosis of the femoral head: A bibliometric analysis. BioMed Res Int (2021) 2021:1433684. doi: 10.1155/2021/1433684 34462719 PMC8403054

