# Supplementary material for: Global research landscape and trends of papillary thyroid cancer therapy: a bibliometric analysis
Source: Front Endocrinol (Lausanne). 2023 Sep 19;14:1252389. doi: 10.3389/fendo.2023.1252389 (PMC10546338; doi:10.3389/fendo.2023.1252389)
Supplement: Supplementary file 1 [file DataSheet_1.docx]

**
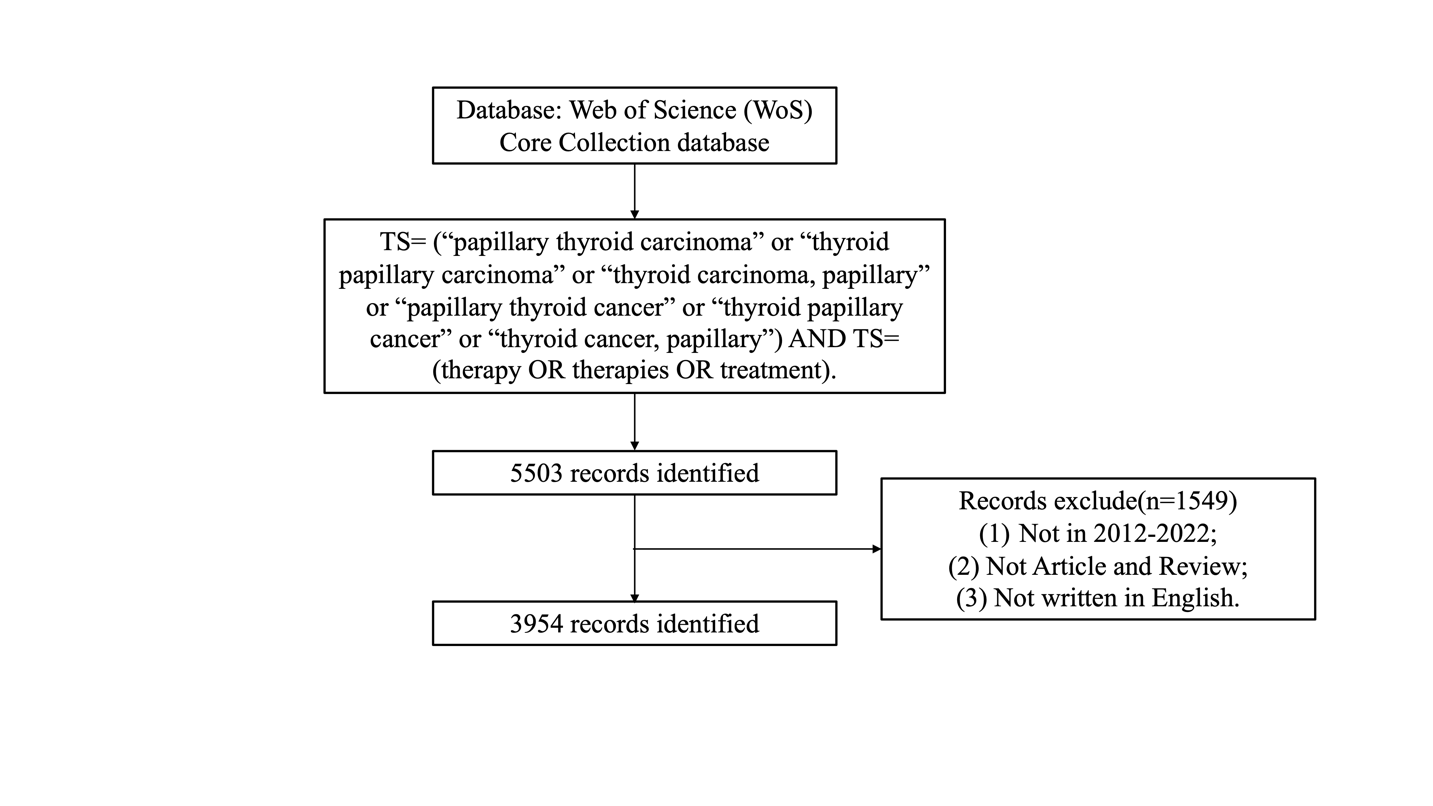
Supplementary FigureS1. Flowchart of data filtration processing and excluding publications.**


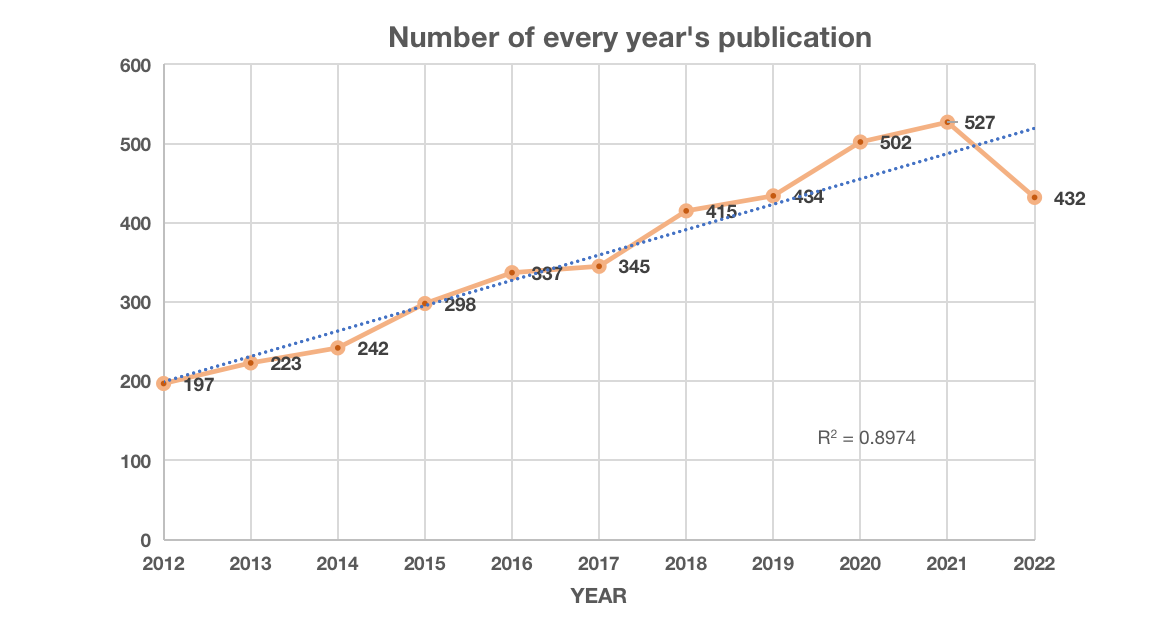


**Supplementary FigureS2. Annual trends of global publications.**


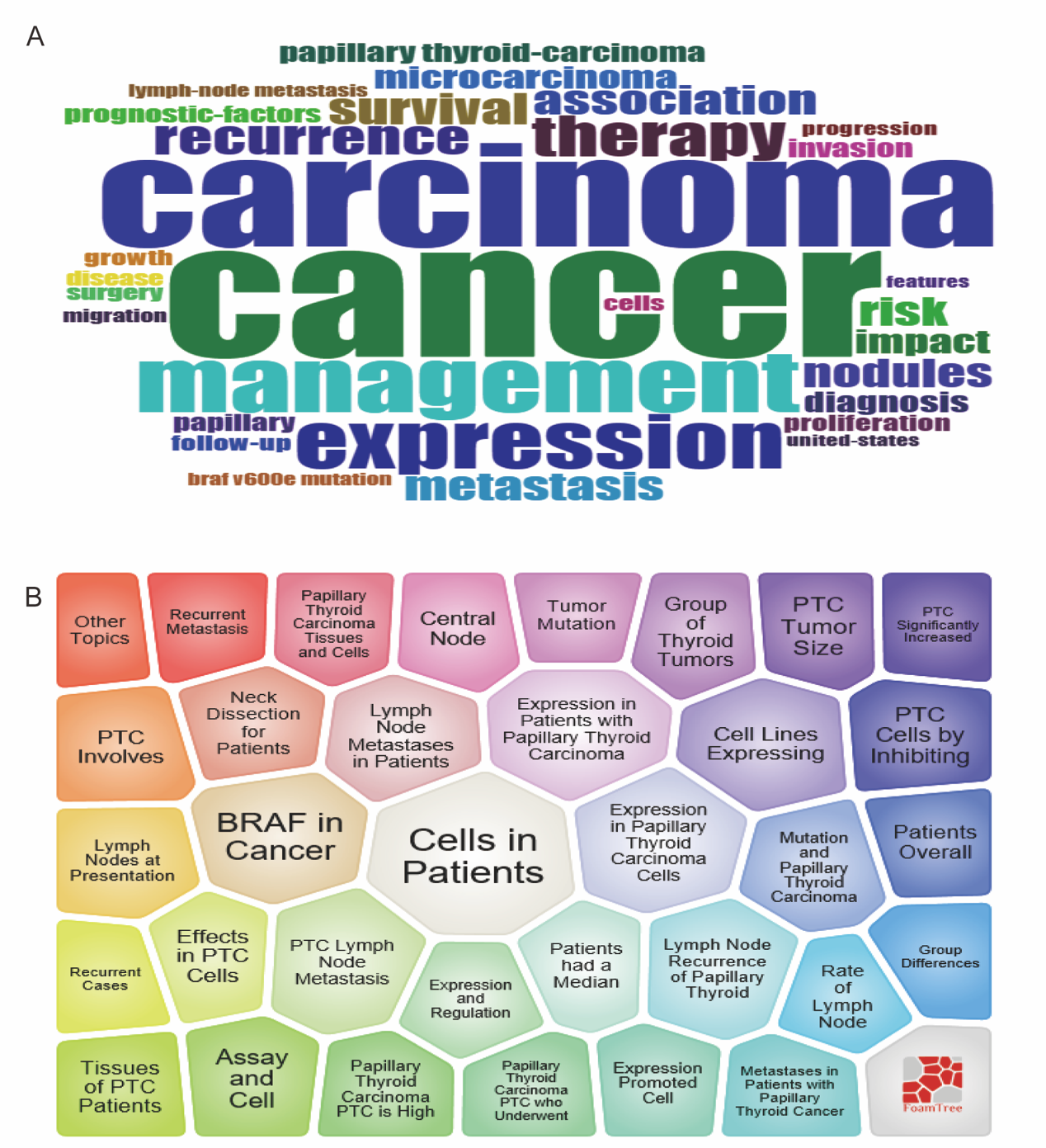


**Supplementary FigureS3**. **(A) keyword tree graph; (B) Topic categories survey for PTC therapy based on the carrot**
